# Multifunctional Exosomes Derived from M2 Macrophages with Enhanced Odontogenesis, Neurogenesis and Angiogenesis for Regenerative Endodontic Therapy: An In Vitro and In Vivo Investigation

**DOI:** 10.3390/biomedicines12020441

**Published:** 2024-02-16

**Authors:** Yujie Wang, Jing Mao, Yifan Wang, Nan Jiang, Xin Shi

**Affiliations:** 1 Center of Stomatology, Tongji Hospital, Tongji Medical College, Huazhong University of Science and Technology, Wuhan 430030, China; wyj_mio@163.com (Y.W.); maojing@hust.edu.cn (J.M.); franc1996@126.com (Y.W.); 2 School of Stomatology, Tongji Medical College, Huazhong University of Science and Technology, Wuhan 430030, China; 3 Hubei Province Key Laboratory of Oral and Maxillofacial Development and Regeneration, Wuhan 430022, China; 4 Central Laboratory, National Engineering Laboratory for Digital and Material Technology of Stomatology, Beijing Key Laboratory of Digital Stomatology, Peking University School and Hospital of Stomatology, Beijing 100081, China; nanjiang@bjmu.edu.cn

**Keywords:** M2 macrophages, exosomes, regenerative endodontic therapy, odonto/osteogenesis, neurogenesis, angiogenesis

## Abstract

Introduction: Exosomes derived from M2 macrophages (M2-Exos) exhibit tremendous potential for inducing tissue repair and regeneration. Herein, this study was designed to elucidate the biological roles of M2-Exos in regenerative endodontic therapy (RET) compared with exosomes from M1 macrophages (M1-Exos). Methods: The internalization of M1-Exos and M2-Exos by dental pulp stem cells (DPSCs) and human umbilical vein endothelial cells (HUVECs) was detected by uptake assay. The effects of M1-Exos and M2-Exos on DPSC and HUVEC behaviors, including migration, proliferation, odonto/osteogenesis, neurogenesis, and angiogenesis were determined in vitro. Then, Matrigel plugs incorporating M2-Exos were transplanted subcutaneously into nude mice. Immunostaining for vascular endothelial growth factor (VEGF) and CD31 was performed to validate capillary-like networks. Results: M1-Exos and M2-Exos were effectively absorbed by DPSCs and HUVECs. Compared with M1-Exos, M2-Exos considerably facilitated the proliferation and migration of DPSCs and HUVECs. Furthermore, M2-Exos robustly promoted ALP activity, mineral nodule deposition, and the odonto/osteogenic marker expression of DPSCs, indicating the powerful odonto/osteogenic potential of M2-Exos. In sharp contrast with M1-Exos, which inhibited the neurogenic capacity of DPSCs, M2-Exos contributed to a significantly augmented expression of neurogenic genes and the stronger immunostaining of Nestin. Consistent with remarkably enhanced angiogenic markers and tubular structure formation in DPSCs and HUVECs in vitro, the employment of M2-Exos gave rise to more abundant vascular networks, dramatically higher VEGF expression, and widely spread CD31^+^ tubular lumens in vivo, supporting the enormous pro-angiogenic capability of M2-Exos. Conclusions: The multifaceted roles of M2-Exos in ameliorating DPSC and HUVEC functions potentially contribute to complete functional pulp–dentin complex regeneration.

## 1. Introduction

The tooth comprises enamel, dentin, pulp, and cementum, the core of which is represented by a pulp–dentin complex. As the hard tissue that encases the pulp, dentin is connected to pulp tissue via odontoblasts and plays a pivotal role in protecting the pulp from damage. On the other hand, the pulp contains blood vessels, nerves, and loose connective tissue and serves multiple essential functions, such as nutrition, sensation, immunity, and defense, which are critical for maintaining tooth vitality. Hence, it is pivotal to preserve the integrity of the pulp–dentin complex for proper tooth function [1]. Caries, periodontitis, and trauma can destroy the pulp–dentin complex, of these, bacterial invasion is the most common cause. In severe cases, they will lead to acute pulpitis or pulp necrosis, which directly affects patients’ physical and mental health and reduces their quality of life [2]. Although conventional root canal treatment can eliminate pain, terminate inflammation, and preserve the affected teeth, it cannot restore the structures and biological functions of the pulp–dentin complex and is, therefore, not an ideal treatment modality [3]. Based on the principles of tissue engineering, Murray et al. [4] proposed the concept of RET in 2007. Dedicated to the structural regeneration and functional reconstruction of the pulp–dentin complex, RET aims to restore tubular dentin and pulp vascular distribution, the innervation network, and immune defense. In this context, RET is expected to be a desirable therapeutic strategy for pulpitis and pulp necrosis.

Emerging as multifunctional cells in the human immune system, macrophages are capable of responding to microenvironmental signals and regulating the body’s immune response under physiological and pathological conditions [5]. Macrophages can be polarized into pro-inflammatory cytotoxic M1 macrophages, in response to lipopolysaccharide (LPS) and interferon-γ (IFN-γ), or into anti-inflammatory reparative M2 macrophages, when confronted with interleukin-4 (IL-4) and IL-13 [6,7]. From a functional point of view, M1 macrophages primarily secrete pro-inflammatory cytokines, such as tumor necrosis factor-α (TNF-α), IL-1, IL-6, and inducible nitric oxide synthase (iNOS), to exacerbate and prolong inflammation. In sharp contrast, M2 macrophages are mainly devoted to inhibiting inflammation responses and promoting tissue repair and regeneration by secreting anti-inflammatory molecules, such as IL-10, arginase, and transforming growth factor-β (TGF-β) [8]. According to various studies, anti-inflammatory factors and bioactive compounds derived from M2 macrophages contributed to osteogenic differentiation [9] and vascularization [10] and had enormous potential for the treatment of nerve injury [11]. Notably, the regulation and polarization of macrophages also played crucial roles in the development of pulpitis and apical periodontitis [12]. However, the effects and exact mechanisms of reparative M2 macrophages in enhancing the repair and regeneration of dental pulp tissue remain to be elucidated.

Exosomes are extracellular vesicles with diameters ranging from 40 to 160 nm, which distinguish them from microvesicles and apoptotic bodies. They are initially produced by the invagination of the cytoplasmic membrane and are then released extracellularly by the fusion of intracellular multivesicular bodies containing intraluminal vesicles with the plasma membrane [13]. Exosomes can be secreted by almost all cells and extensively distributed in various body fluids. Once taken up by target cells, exosomes mediate intercellular communication and modulate a wide range of physiopathological microenvironments by delivering functional factors, such as nucleic acids, proteins, or lipids [14,15]. In recent years, due to unique, desirable advantages, such as diverse sources, low immunogenicity, inherent targeting, and high safety, exosomes have been gradually exploited as an innovative strategy for remodeling the regenerative microenvironment and achieving the regeneration of a variety of oral tissues, including pulp tissue [16,17,18,19]. Li et al. [20] have demonstrated that M2-Exos could promote osteogenesis and inhibit adipogenesis in vitro. By employing murine periodontitis models, Chen et al. [21] illustrated that M2-Exos regulated cell differentiation and bone metabolism by activating the IL-10/IL-10R signaling pathway in bone marrow stromal cells and bone marrow macrophages, consequently suppressing osteoclastogenesis and facilitating alveolar bone formation. In addition, as indicated by Huang et al. [22], M2-Exos effectively stimulated neurological recovery and angiogenesis after spinal cord injury. Hence, representing a new idea, M2-Exos hold tremendous promise for inducing tissue repair and regeneration.

Taken together, this study intends to explore the feasibility of utilizing M2-Exos in ameliorating the biological functions of DPSCs and HUVECs and promoting the regeneration of the pulp–dentin complex in vitro and in vivo, in an attempt to provide support for the development of RET and accelerate the clinical translation of regenerative endodontic practice.

## 2. Materials and Methods

The present study was approved by the Medical Ethics Committee (No. TJ-IRB202112103) and Laboratory Animal Welfare and Ethics Committee (No. TJH-202301004) of Tongji Hospital, Tongji Medical College, Huazhong University of Science and Technology.

### 2.1. Isolation and Characterization of DPSCs

After obtaining informed consent, healthy orthodontic or wisdom teeth extracted from patients aged 12–18 years were collected at the Department of Oral and Maxillofacial Surgery, Tongji Hospital, Tongji Medical College, Huazhong University of Science and Technology. Then, we separated the dental crown from the root under aseptic conditions. To harvest DPSCs, the pulp tissue was isolated from the pulp cavity, chopped into small fragments, and digested in 3 mg/mL collagenase type I (BioFroxx, Einhausen, Germany) and 4 mg/mL dispase (BioFroxx, Einhausen, Germany) for 90 min at 37 °C. After digestion, the supernatant was discarded by centrifugation, and the cell suspensions were inoculated in an α-modified minimum essential medium (α-MEM) (Gibco, Waltham, MA, USA) supplemented with 10% fetal bovine serum (FBS) (Keyi, Wuhan, China) at 37 °C under 5% CO_2_.

To characterize the obtained DPSCs, the primary cells were cultured to passages 3–5 (P3–5) and their osteogenic and adipogenic differentiation was induced. On the 21st day of osteogenic induction (Cyagen, Guangzhou, China), the culture medium was aspirated. Subsequently, the cells were washed twice with phosphate-buffered saline (PBS) (BioSharp, Hefei, China), fixed with 4% paraformaldehyde (PFA) (Servicebio, Wuhan, China) for 30 min, and stained with 0.1% alizarin red S solution (Solarbio, Beijing, China) at room temperature for 30 min. The red mineralized nodules were observed using an inverted microscope (Olympus, Tokyo, Japan). On the 21st day of adipogenic induction (Cyagen, Guangzhou, China), the fixed cells were stained with oil red O solution (Cyagen, Guangzhou, China) for 30 min at room temperature, and the formed lipid particles were observed under a microscope. In addition, the P3–5 DPSCs were digested by trypsin (Gibco, Waltham, MA, USA) and cultured in centrifuge tubes containing chondrogenic differentiation medium (Cyagen, Guangzhou, China). On the 21st day of induction, the cartilage balls were collected, fixed, sectioned, and stained with toluidine blue for microscopic observation. Next, the immunophenotyping characterization of the P3–5 DPSCs was identified by flow cytometry using antibodies against CD34-PE, CD73-APC, CD90-FITC, and CD105-PC5.5 (BD Science, San Jose, CA, USA). All antibodies were employed at a dilution of 1: 500. The antibodies were gently mixed and incubated for 30 min at room temperature, then determined utilizing CytoFLEX flow cytometry (Beckman Coulter, Miami, FL, USA).

### 2.2. Macrophage Polarization and Identification

Human monocytic THP-1 cells were purchased from HyCyte and cultured in a special medium (HyCyte, Suzhou, China). The cells were differentiated into macrophages (M0) by 24 h incubation with 100 nM phorbol 12-myristate 13-acetate (PMA) (Lianke, Hangzhou, China). Subsequently, the matured M0s were incubated in a medium supplemented with 100 ng/mL LPS (PeproTech, Rocky Hill, NJ, USA) and 20 ng/mL IFN-γ (PeproTech, Rocky Hill, NJ, USA) for 48 h until they were polarized into M1 macrophages. On the other hand, M2 macrophage polarization was achieved by incubating M0 with 20 ng/mL IL-4 (PeproTech, Rocky Hill, NJ, USA) and 20 ng/mL IL-13 (PeproTech, Rocky Hill, NJ, USA) for 48 h.

The mRNA expression of M1-correlated markers *iNOS*, *IL-6*, *CD86*, *TNF-α*, and the M2-specific markers, including *insulin-like growth factor* (*IGF*), *TGF-β*, *VEGF*, and *CD163,* were analyzed by real-time quantitative polymerase chain reaction (RT-qPCR) analysis. First, the total RNA in M1 and M2 was extracted employing a commercialized RNA extraction kit (Accurate, Changsha, China) following the manufacturer’s instructions, and the quantity and quality of the harvested total RNA were assessed with a microvolume spectrophotometer (Kaiao, Beijing, China). Then, cDNA was synthesized using a reverse transcription kit (Accurate, Changsha, China), and the reaction system was listed in Table S1. The thermocycler was set as follows: 37 °C, 15 min; 85 °C, 5 s. Next, RT-qPCR was performed using a SYBR Green master mix (Accurate, Changsha, China) on an LC480II light thermal cycler (Roche, Basel, Switzerland). The primer sequences are presented in Table S2, and the reaction system is shown in Table S3. The thermal cycler program involved an initial hot start step of 20 min at 95 °C, followed by 40 cycles of 95 °C for 3 s and 60 °C for 30 s. The experiment was repeated more than three times. To confirm the primer specificity, melting curve analysis was performed after each amplification. Amplification signals were normalized by *β-actin* as the housekeeping gene. The relative quantification and fold changes of gene expression were evaluated by the 2^−ΔΔCt^ method.

To conduct immunofluorescence staining, THP-1 cells were inoculated at 1 × 10^5^ cells/well in 24-well plates containing glass coverslips, and M1 and M2 polarization were induced as described above. After polarization, cells were washed with pre-cooled PBS, fixed with 4% PFA for 20 min, and washed three times with the permeabilizing solution (Beyotime, Nanjing, China) on a shaker. Blocking was performed at 37 °C for 1 h with 5% milk solution (Servicebio, Wuhan, China) in PBS. The cells were then incubated with the primary antibodies CD68, iNOS, and CD206 (1:500) (ABclonal, Wuhan, China) overnight at 4 °C, rinsed several times with PBS, and incubated with fluorophore-conjugated goat anti-rabbit secondary antibody (1: 100) (ABclonal, Wuhan, China) for 1 h at room temperature. After washing, the cells were stained with DNA-specific blue fluorescent DAPI (Solarbio, Beijing, China) for 5 min, and the coverslips were mounted on glass slides and observed under a laser confocal microscope (Zeiss, Jena, Germany).

### 2.3. Exosome Extraction and Characterization

After successful polarization of M1 and M2 macrophages, the medium was changed to serum-free RPMI 1640 medium (Gibco, Waltham, MA, USA), and the M1 and M2 supernatants were collected after 48 h of incubation and stored at 4 °C. The supernatants were transferred into 50 mL centrifuge tubes and centrifuged in a high-speed low-temperature centrifuge (Eppendorf, Hamburg, Germany) at 2000× *g* for 30 min at 4 °C to remove cells and impurities. Then, the newly obtained supernatants were loaded into new 50 mL centrifuge tubes and centrifuged at 10,000× *g* at 4 °C for 30 min to remove cellular debris. Subsequently, the supernatants were collected and slowly transferred to 26.3 mL Beckman ultracentrifuge tubes and centrifuged at 100,000× *g* at 4 °C for 90 min using an ultracentrifuge (Beckman Coulter, Miami, FL, USA) to obtain exosomes. Immediately after centrifugation, all supernatant liquid was carefully and completely discarded, and the precipitated pellets at the bottom of centrifuge tubes were rinsed repeatedly with 100 μL PBS. Finally, the M1-Exos and M2-Exos were resuspended in PBS and stored at −80 °C for later use.

A BCA assay was administered to evaluate the total protein concentration of exosomes according to the manufacturer’s instructions. Then, 15 μL M1-Exos and M2-Exos stock solutions were pipetted and diluted to 200 μL with sterile PBS. A total of 300 μL of double-distilled water was applied to repeatedly wash the sample wells of the nano-particle meter (Particle Metrix, Meerbusch, Germany). Next, the diluted M1-Exos and M2-Exos were injected into the sample wells. Lastly, the M1-Exos and M2-Exos were subjected to nanoparticle tracking analysis (NTA) for the detection of exosome concentration and size distribution. To examine the morphology of exosomes, M1-Exos and M2-Exos were added dropwise onto a copper grid with 2% phosphotungstic acid solution (Solarbio, Beijing, China), and dried at room temperature for 5 min. The ultrastructure of M1-Exos and M2-Exos was observed using a transmission electron microscope (TEM) (Hitachi, Tokyo, Japan). Aiming to validate the presence of characteristic exosome marker proteins, we carried out a western blot (WB) analysis for M1-Exos and M2-Exos to determine CD63 and TSG101.

### 2.4. Exosome Labeling and Cellular Uptake

A total of 500 μg of M1-Exos and M2-Exos were labeled with the lipophilic dye PKH-26 (Umibio, Shanghai, China), respectively, followed by ultracentrifugation to remove excess dye. Then, the PKH-26-labeled exosomes were added to DPSCs or commercially obtained HUVECs (iCell, Shanghai, China) and incubated for 24 h. Afterward, the co-incubated cells were washed three times with PBS and subsequently fixed with PFA for 20 min. Next, fluorescent phalloidin (Abbkine, Wuhan, China) was employed to label the actin filament of the cytoskeleton in DPSCs and HUVECs for 30 min, and DAPI (Solarbio, Beijing, China) was utilized for staining cell nuclei for 5 min. Finally, the cells were rinsed three times with PBS and photographed under a laser confocal microscope.

### 2.5. Cell Proliferation and Migration

A CCK-8 assay was conducted to investigate the effect of M1-Exos and M2-Exos on the proliferative potential of DPSCs and HUVECs. The P3–5 DPSCs and HUVECs were separately seeded into 96-well plates at a density of 5 × 10^3^ cells/well, and each type of cell was divided into three groups as follows: (1) control; (2) 50 μg/mL M1-Exos; (3) 50 μg/mL M2-Exos. More than three replicate wells were set up for each group. After 1, 3, and 5 days of incubation, the culture medium was replaced with the serum-free medium containing 10% CCK-8 solution (*v*/*v*) (MedChemExpress, Monmouth Junction, NJ, USA). Soon after incubation at 37 °C in the dark for 2 h, the optical density (OD) was recorded at 450 nm using an enzyme labeling instrument (Molecular Devices, San Jose, CA, USA). The ODs of the blank wells (culture medium and CCK-8) were also recorded.

The role of 50 μg/mL exosomes in cell migration capacity was detected by a transwell assay. A total of 4 × 10^5^ cells/well of DPSCs and HUVECs were separately plated in the upper chamber of 24-transwell plates (Corning, New York, NY, USA) with 200 μL serum-free medium, and a culture medium containing 50 μg/mL M1-Exos or M2-Exos was incorporated into the lower chamber. An equal volume of culture medium without exosomes served as a control group. Each group possessed more than three replicate wells. After incubation for 24 h at 37 °C, the cells remaining on the upper layer of the membrane were gently wiped off with a cotton swab, while the cells that had migrated through the membrane were fixed with 4% PFA for 30 min and stained with 0.4% crystal violet (Sigma-Aldrich, Saint Louis, CA, USA) for 15 min. Three fields of view were randomly chosen from each membrane to capture images. The number of cells per field was counted for statistical analysis.

### 2.6. DPSC Odonto/Osteogenic Differentiation Detection

Following the experimental design mentioned above, DPSCs were cultured in an osteogenic induction medium (Cyagen, Guangzhou, China) supplemented with/without 50 μg/mL exosomes. 5 days later, the total RNA was extracted and RT-qPCR was performed to detect the expression of *ALP*, *DSPP*, *collagen type I α 1* (*COL-1α1*), *dentin matrix protein-1* (*DMP-1*), *osteocalcin* (*OCN*), *bone morphogenetic protein-2* (*BMP-2*), and *runt-related transcription factor-2* (*RUNX-2*).

To implement ALP staining and an ALP activity assay, DPSCs were cultured in an osteogenic induction medium with/without exosomes for 5 days and then fixed with 4% PFA for 30 min. The ALP staining solution was prepared according to the instructions of an ALP kit (Beyotime, Nanjing, China). Then, the fixed DPSCs were incubated with the ALP staining solution for 60 min at room temperature, washed to remove the excess staining solution, and air-dried. In the end, the resultant staining was visualized under a microscope, and the blue color was regarded as a positive staining. To determine ALP activity, total protein was extracted from cells and analyzed using an ALP protein quantitative analysis kit (Jiancheng, Nanjing, China) following the manufacturer’s protocol, and the OD value at 520 nm was estimated.

For alizarin red S staining and semi-quantitative measurement, DPSCs were induced by an osteogenic medium (Cyagen, Guangzhou, China) mixed with/without exosomes for 3 weeks, and alizarin red S staining was performed as aforementioned to observe red mineralized nodules. Subsequently, 10% cetylpyridinium chloride (Yuanye, Shanghai, China) was used to dissolve the mineral nodules, and the calcium concentration was detected by OD value at 562 nm.

### 2.7. DPSC Neurogenic Differentiation Assay

According to the experimental design, DPSCs were cultured in the neurogenic induction medium (Puhe, Wuxi, China) with/without 50 μg/mL exosomes. 5 days later, total RNA was extracted, and RT-qPCR was performed to detect the expression level of *Nestin*, *glial cell-derived neurotrophic factor* (*GDNF*), and *brain-derived neurotrophic factor* (*BDNF*).

For immunofluorescence staining, DPSCs were seeded into 12-well plates containing glass coverslips. After 5 days, the cells were incubated with primary antibody Nestin (1: 100) (Abclonal, Wuhan, China), followed by treatment with fluorophore-conjugated secondary antibody. Lastly, the cells were stained with phalloidin for 30 min and stained with DAPI for 5 min. The neurogenic property was assessed by a semi-quantitative analysis of fluorescence intensity.

### 2.8. In Vitro Angiogenesis Assay

Based on the above experimental design, DPSCs and HUVECs were incubated in an angiogenic induction medium (iCell, Shanghai, China) with/without exosomes, respectively. 5 days later, total RNA was isolated and RT-qPCR was conducted to reveal the expression of angiogenic markers, including *VEGF*, *angiopoietin II* (*ANG II*), and *platelet-derived growth factor A* (*PDGFA*).

To explore the tube formation potential of DPSCs and HUVECs, Matrigel (ABW Mogengel, Xiamen, China) was added to 96-well plates under ice bath conditions and then incubated at 37 °C for 1 h until the Matrigel solidified into a gel. DPSCs and HUVECs were cultured in an angiogenic medium (iCell, Shanghai, China) conditioned with/without exosomes for 3 days. Afterward, single-cell suspensions of DPSCs and HUVECs were prepared and plated into 96-well plates with Matrigel at a density of 1 × 10^4^ cells/well. Approximately 4–6 h later, images of the tube-like structures were taken. The angiogenic property was assessed by measuring the number of nodes, the number of branches, the number of junctions, the total length, the total length of branches, and the total length of master segments from three random microscopic fields using Image J software (Version 1.53t, National Institutes of Health, Bethesda, MD, USA). Each assay was repeated at least three times.

### 2.9. In Vivo Angiogenesis Assay

A total of 500 μL Matrigel mixed with exosomes or cells was injected subcutaneously into the backs of 8-week-old BALB/c nude mice (Gempharmatech, Nanjing, China). Mice were randomly assigned to four groups depending on cell type (*n* = 3): (1) PBS; (2) 1 × 10^7^ DPSCs or HUVECs; (3) M1-Exos and 1 × 10^7^ DPSCs or HUVECs; (4) M2-Exos and 1 × 10^7^ DPSCs or HUVECs. The sample size was estimated based on a previous study [23]. All mice were cared for by qualified breeders and highly skilled veterinarians at a specific pathogen-free animal center. After implantation for 2 weeks, the mice were euthanized by exposure to CO_2_. Then, the Matrigel plugs were dissected and fixed with 4% PFA, embedded in paraffin, and sectioned into slides. For immunofluorescence staining, the plug sections were incubated with the primary antibody VEGF (1:100) (Abclonal, Wuhan, China), followed by treatment with a fluorophore-conjugated secondary antibody. For immunohistochemical staining, the Matrigel plug sections were incubated with the primary antibody CD31 (1:100) (Abclonal, Wuhan, China), followed by a secondary antibody. The angiogenic property was evaluated by semi-quantitative analysis of the fluorescence intensity of VEGF and the number of CD31^+^ tubular structures. In the present study, animals with inferior status, such as sickness, injury, or weight loss greater than 20%, were subjected to euthanasia. No mice were excluded from this study.

### 2.10. Statistical Analysis

All experiments were repeated at least three times. Statistical analysis was processed using GraphPad Prism software (Version 9.0.0, San Diego, CA, USA). The results were expressed as mean ± SD. Student’s *t*-test was applied for two-group statistical comparisons and one-way analysis of variance (ANOVA) followed by Tukey’s test was performed to compare three or four groups. *p* < 0.05 was considered statistically significant (* *p* < 0.05, ** *p* < 0.01, *** *p* < 0.001, **** *p* < 0.0001).

## 3. Results

### 3.1. DPSC Culture and Characterization

**Figure 1:**
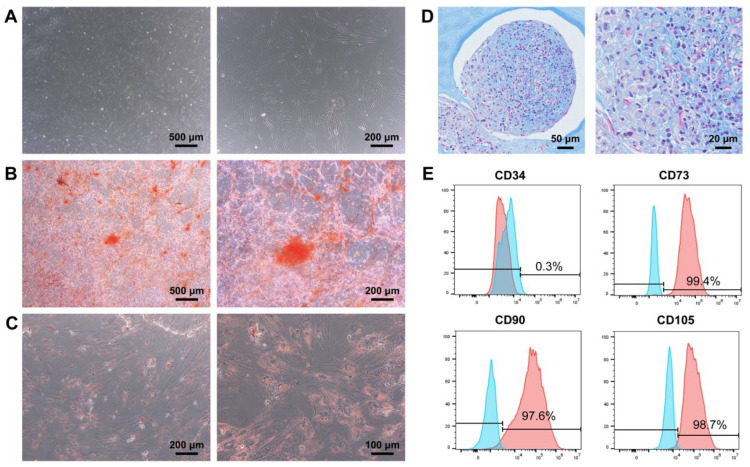
DPSCs extracted from human permanent teeth exhibit MSC attributes. (**A**) Representative image of P4 DPSCs, which acquired an elongated spindle morphology. Scale bars, 500 μm and 200 μm (high magnification). (**B**–**D**) DPSCs elicited robust multipotency for osteogenesis, adipogenesis, and chondrogenesis. (**B**) DPSCs exhibited osteogenic differentiation potential, as revealed by orange–red mineral nodule deposition. Scale bars, 500 μm and 200 μm (high magnification). (**C**) DPSCs showed adipogenic differentiation capacity with the formation of intracellular lipid droplets. Scale bars, 200 μm and 100 μm (high magnification). (**D**) DPSCs possessed chondrogenic differentiation ability, as indicated by the extracellular accumulation of glycosaminoglycans. Scale bars, 50 μm and 20 μm (high magnification). (**E**) Flow cytometry suggested that apart from negative marker CD34, positive markers CD73, CD90, and CD105 were highly expressed in DPSCs. Blue area: Isotype control; Red area: Antibody staining.

Under a light microscope, it could be seen that the isolated P4 DPSCs adhered to the well surface and exhibited an elongated spindle-shaped morphology, ensuring a healthy state (Figure 1A). To determine the multilineage differentiation potential of DPSCs, we performed osteogenic, adipogenic, and chondrogenic induction experiments. After culturing DPSCs in the osteogenic inductive medium for 3 weeks, massive orange–red mineralized nodules could be observed by alizarin red S staining, indicative of the morphologic differentiation of DPSCs into osteoblasts (Figure 1B). When cultured in the adipogenic inductive medium for 3 weeks, DPSCs were capable of differentiating to adipocytes with noticeable intracellular red lipid droplets, as revealed by oil red O staining (Figure 1C). Furthermore, in response to chondrogenic induction, DPSCs gave rise to remarkable extracellular accumulations of cartilage-like glycosaminoglycans that were positively blue by toluidine blue staining, demonstrating the differentiation of functional chondrocytes from DPSCs (Figure 1D). In addition, according to flow cytometry analysis, the DPSCs lacked the expression of hematopoietic stem cell-associated marker CD34 but highly expressed crucial mesenchymal stem cell (MSC)-related surface markers, including CD73, CD90, and CD105 (Figure 1E). Together, the above results suggested that the harvested DPSCs possessed the characteristics of MSCs, thus laying a cellular foundation for the subsequent implementation of this study.

### 3.2. Macrophage Polarization and Validation

**Figure 2:**
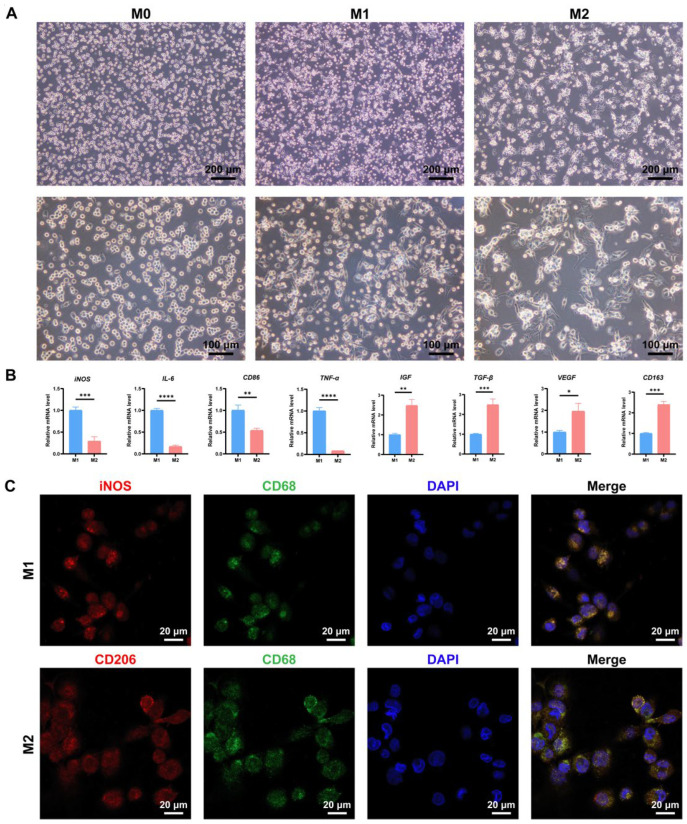
M0 macrophages derived from THP-1 monocytes can be polarized into M1 and M2 macrophages. (**A**) Representative images of M0, M1, and M2 macrophages as recorded using a light microscope. M0 macrophages were characterized by plastic adherence and small round shape without cellular extensions; M1 macrophages exhibited larger size and elongated shape with delicate cytoplasmic projections; M2 macrophages displayed abundant cytoplasmic protrusions and diverse morphologies. Scale bars, 200 μm and 100 μm (high magnification). (**B**) RT-qPCR was conducted to verify the expression of mRNAs associated with M1 and M2 macrophages. *iNOS*, *IL-6*, *CD86*, and *TNF-α* were considerably increased in M1 macrophages, whereas the levels of *IGF*, *TGF-β*, *VEGF,* and *CD163* were remarkably higher in M2 than in M1. (**C**) Representative immunofluorescence staining of M1 and M2 macrophages. In the upper panel, M1 macrophages were identified by the co-expression of the M1-specific marker iNOS (red fluorescence), pan-macrophage marker CD68 (green fluorescence), and nuclear marker DAPI (blue fluorescence); in the lower panel, the co-expression of the M2-specific marker CD206 (red fluorescence), CD68 (green fluorescence), and DAPI (blue fluorescence) was administered to symbolize M2 macrophages. Scale bar, 20 μm. * *p* < 0.05, ** *p* < 0.01, *** *p* < 0.001, **** *p* < 0.0001.

PMA has been commonly utilized to initiate the differentiation of human monocytic THP-1 cells. In our study, following induction with 100 nM PMA for 24 h, the suspended THP-1 cells obtained a macrophage-like appearance characterized by plastic adherence and a small, round, translucent structure lacking cellular extensions (Figure 2A), indicating that THP-1 monocytes matured and differentiated into M0 macrophages. Subsequently, aimed at facilitating the polarization of M1 macrophages, the differentiated M0 were exposed to 100 ng/mL LPS and 20 ng/mL IFN-γ for 48 h. When examined using a light microscope, changes in cellular size and morphology were noticed, with cells exhibiting larger size and an elongated shape, as well as containing delicate cytoplasmic projections (Figure 2A), which preliminarily suggested that M0 polarized into M1 macrophages. Astonishingly, after stimulation in the presence of 20 ng/mL IL-4 and 20 ng/mL IL-13, macrophages were distinguished by their abundant cytoplasmic protrusions and diverse morphologies, including round shape, elongated shape, and spindle-like shape (Figure 2A), implying that M0 macrophages were polarized towards the phenotypes resembling M2. To further verify the polarization of M1 and M2, we then performed RT-qPCR. Importantly, compared with M2 phenotypes, the expression of pro-inflammatory cytokines, including *iNOS*, *IL-6*, *CD86*, and *TNF-α* was considerably increased in M1 macrophages (Figure 2B). In contrast, a significantly enhanced expression of anti-inflammatory factors, such as *IGF*, *TGF-β*, *VEGF,* and *CD163,* was detected in M2 rather than M1 (Figure 2B), supporting favorable polarization of macrophages. Consistent with these findings, immunofluorescence staining validated that the polarized M1 co-expressed specific marker iNOS and pan-macrophage marker CD68 (Figure 2C, upper panel), while the co-expression of representative marker CD206 and pan-macrophage marker CD68 was evidenced in the polarized M2 macrophages (Figure 2C, lower panel). Hence, under appropriate stimulating conditions in the present study, M0 macrophages derived from THP-1 cells could be polarized into M1 and M2 macrophages.

### 3.3. Exosome Isolation, Characterization, and Cellular Uptake Assay

**Figure 3:**
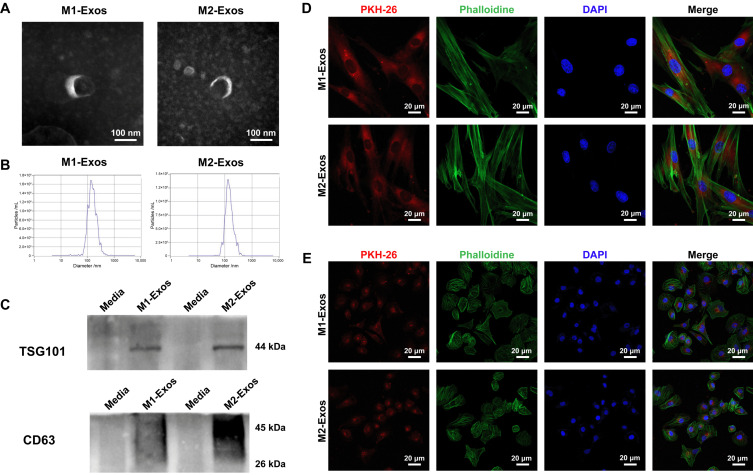
Exosome characterization and uptake assay. (**A**–**C**) M1-Exos and M2-Exos separated by differential centrifugation were characterized by TEM, NTA, and WB. (**A**) Under TEM imaging, M1-Exos and M2-Exos displayed a typical round cup-shaped structure with a diameter of 100 nm. Scale bar, 100 nm. (**B**) As validated by NTA, M1-Exos and M2-Exos had an average diameter of around 140 nm. (**C**) Exosome-specific markers TSG101 and CD63 were highly expressed in M1-Exos and M2-Exos when assessed with WB. (**D**,**E**) The uptake of exosomes by DPSCs and HUVECs. (**D**) The co-immunostaining of exosomes (red fluorescence), actin filament (green fluorescence), and nucleus (blue fluorescence) confirmed that M1-Exos (upper panel) and M2-Exos (lower panel) were evenly distributed around the cytoplasm and nucleus in DPSCs. Scale bar, 20 μm. (**E**) M1-Exos (red fluorescence, upper panel) and M2-Exos (red fluorescence, lower panel) were internalized by HUVECs and scattered in the cytoplasm (green fluorescence) around the nucleus (blue fluorescence). Scale bar, 20 μm.

Exosomes were collected from the supernatants of M1 and M2 macrophages using differential centrifugation, respectively. After the protein concentration of exosomes was determined by BCA assay, the existence of exosomes was characterized by TEM, NTA, and WB (Figure 3A–C). As identified by TEM, both M1-Exos and M2-Exos exhibited characteristic concave or cup-shaped morphologies with diameters of around 100 nm (Figure 3A). Complementary to TEM assessment, NTA revealed that at a concentration of 8.0 × 10^10^ particles/mL, 95.2% of M1-Exos were distributed between 106.2 and 167.5 nm in diameter (Figure 3B), and the average diameter was 147.1 nm. For M2-Exos, the concentration was measured to be 5.8 × 10^10^ particles/mL. The diameter distribution of 95.5% of M2-Exos ranged between 79.9 and 162.5 nm (Figure 3B), and the average diameter was 140.1 nm, which met the size standard of exosomes. Moreover, according to WB detection, M1-Exos and M2-Exos expressed the exosome-specific markers TSG101 and CD63 (Figure 3C), consistent with previous studies. In summary, the above results indicate that M1-Exos and M2-Exos have been successfully extracted in our study, paving the way for further exploring their biological effects. Next, to confirm cellular internalization of exosomes by DPSCs and HUVECs, M1-Exos and M2-Exos labeled with PKH-26 were incubated with DPSCs and HUVECs for 24 h, respectively. As observed using a laser confocal microscope, numerous M1-Exos (Figure 3D, upper panel) and M2-Exos (Figure 3D, lower panel) stained with red fluorescence were extensively dispersed between the cytoplasm and the nucleus in DPSCs, suggesting the desirable uptake of exosomes. Similarly, M1-Exos (Figure 3E, upper panel) and M2-Exos (Figure 3E, lower panel) were also identified widely in the cytoplasm around the nucleus of HUVECs. In consequence, M1-Exos and M2-Exos could be effectively internalized by both DPSCs and HUVECs, which elicited tremendous potential in modulating the biological functions of the target cells.

### 3.4. Effect of M1-Exos and M2-Exos on Cell Proliferation and Migration

**Figure 4:**
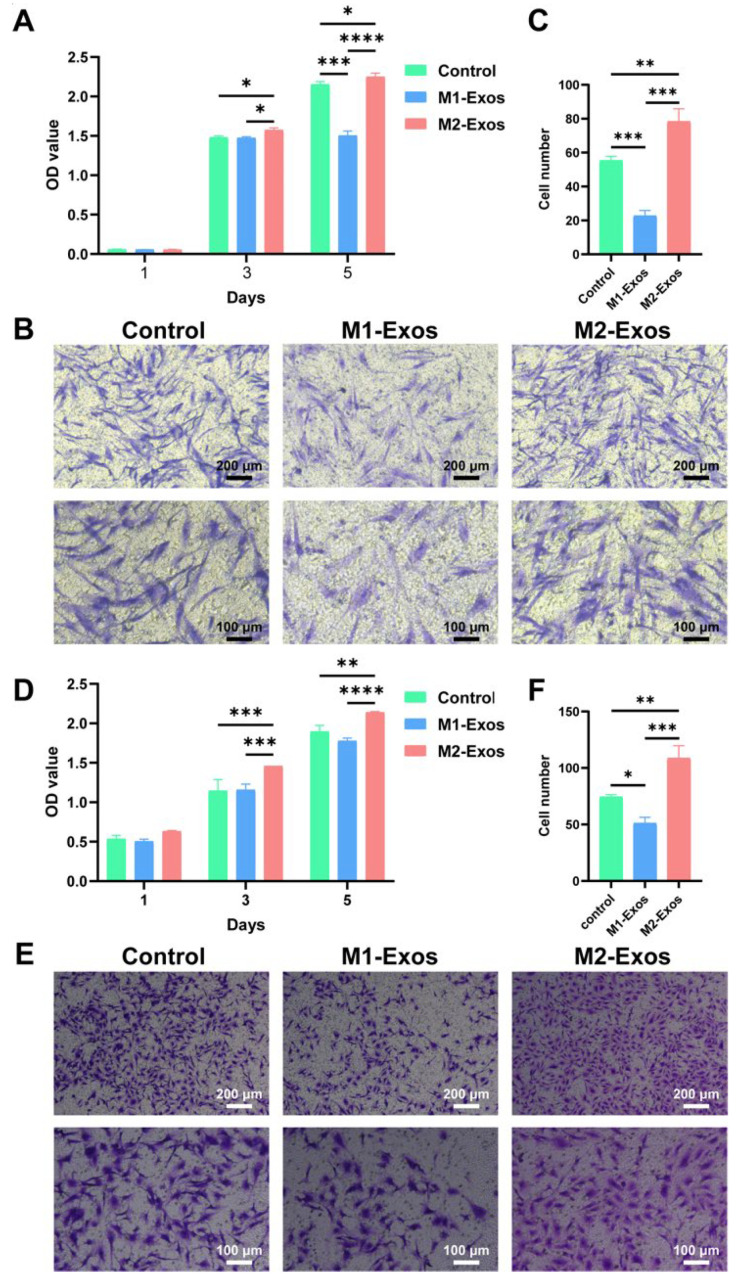
The role of M1-Exos and M2-Exos in cellular proliferation and migration. (**A**,**D**) As revealed by the CCK-8 assay, M2-Exos were capable of fostering the proliferation of DPSCs (**A**) and HUVECs (**D**). Conversely, M1-Exos negatively regulated cellular proliferation. (**B**,**C**,**E**,**F**) According to the transwell assay, as opposed to M1-Exos, which enormously repressed the migratory potential of DPSCs (**B**,**C**) and HUVECs (**E**,**F**), M2-Exos elicited a noticeable promoting effect on their migration. Scale bars, 200 μm and 100 μm (high magnification). * *p* < 0.05, ** *p* < 0.01, *** *p* < 0.001, **** *p* < 0.0001.

To illuminate the impact of two distinct exosomes on the biological functions of DPSCs and HUVECs, we performed a CCK-8 assay and a transwell assay to evaluate the role of 50 μg/mL exosomes in cellular proliferation and migration. The results of the CCK-8 assay showed that M2-Exos dramatically promoted the proliferation of DPSCs (Figure 4A) and HUVECs (Figure 4D) in 3 and 5 days. Although M1-Exos executed no considerably inhibitory effect on the proliferation of HUVECs in 5 days (Figure 4D), a detrimental role of M1-Exos in DPSC proliferation capacity has been witnessed (Figure 4A). As indicated by the transwell assay, in contrast to M1-Exos, which considerably suppressed the migration of DPSCs (Figure 4B,C) and HUVECs (Figure 4E,F), the employment of M2-Exos substantially augmented the number of migrated DPSCs and HUVECs, supporting the beneficial role of M2-Exos in enhancing cell migratory potential. Collectively, compared with M1-Exos, M2-Exos could ameliorate cellular biological behaviors and markedly facilitate the proliferation and migration capability of DPSCs and HUVECs.

### 3.5. Effect of M1-Exos and M2-Exos on Odonto/Osteogenic Differentiation of DPSCs In Vitro

**Figure 5:**
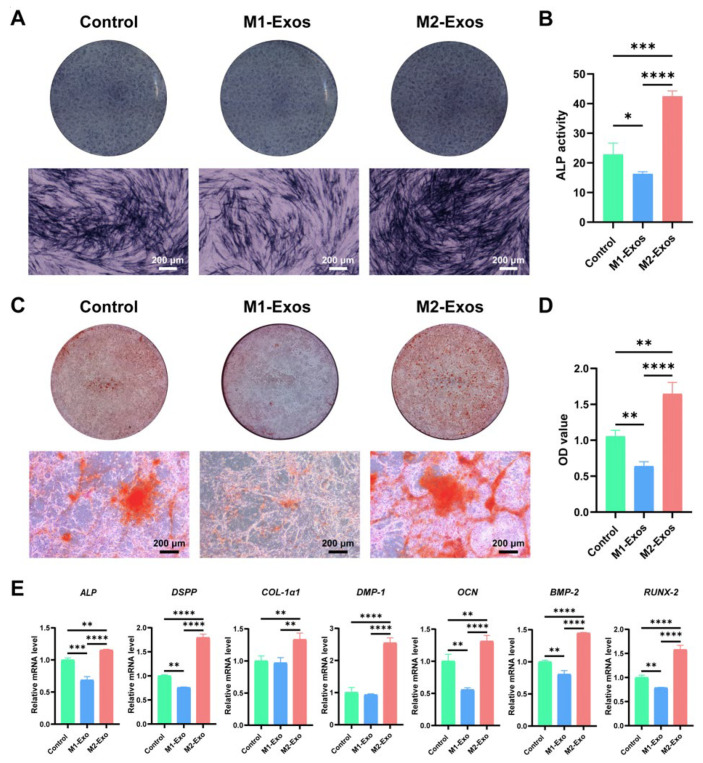
Effect of M1-Exos and M2-Exos on odonto/osteogenic differentiation of DPSCs in vitro. (**A**,**B**) M1-Exos and M2-Exos exhibited distinguishable roles in ALP staining and activity. (**A**) In contrast to M1-Exos, which unfavorably hampered ALP staining, M2-Exos robustly augmented ALP staining. Scale bar, 200 μm. (**B**) M2-Exos exerted a strong enhancing role in ALP activity, whereas M1-Exos extraordinarily repressed ALP activity. (**C**,**D**) M1-Exos and M2-Exos possessed an opposite impact on alizarin red S staining and semi-quantitative analysis. (**C**) According to alizarin red S staining, large amounts of orange–red calcium nodules were determined after the introduction of M2-Exos, but, unfortunately, M1-Exos gave rise to sparse mineral deposits with an attenuated color. Scale bar, 200 μm. (**D**) Semi-quantitative analysis of alizarin red S suggested that compared with M1-Exos, which diminished the OD value, M2-Exos considerably elevated the OD value. (**E**) Similarly, in response to M2-Exos, the mRNA expression of odonto/osteogenic markers, including *ALP*, *DSPP*, *COL-1α1*, *DMP-1*, *OCN*, *BMP-2*, and *RUNX-2* was upregulated remarkably in DPSCs. However, M1-Exos negatively regulated these gene expressions. * *p* < 0.05, ** *p* < 0.01, *** *p* < 0.001, **** *p* < 0.0001.

Aimed at elucidating the impact of M1-Exos and M2-Exos on the odonto/osteogenesis of DPSCs, we carried out a series of experiments, including ALP staining, alizarin red S staining, and RT-qPCR. ALP staining showed that the application of M2-Exos contributed to the extensive accumulation of black–blue cloddy precipitates with high density in the cytoplasm of DPSCs (Figure 5A). However, when challenged with M1-Exos, DPSCs acquired considerably attenuated staining, as demonstrated by granular or lamellar precipitates dispersed in the cytoplasm (Figure 5A). In line with this, M1-Exos exhibited a significantly inhibitory effect on ALP activity (Figure 5B). Nonetheless, on the contrary, M2-Exos remarkably boosted ALP activity (Figure 5B), emphasizing the decisive role of M2-Exos in initiating the odonto/osteogenic differentiation of DPSCs. As clarified by alizarin red S staining, M2-Exos could significantly increase the deposition of orange–red calcium nodules, which were widely distributed in the form of clusters or plaques (Figure 5C). In sharp contrast, the intervention of M1-Exos substantially decreased the formation of mineral nodules, which were scattered in a spot manner, displaying a diminished color (Figure 5C). In accordance with alizarin red S staining, the semi-quantitative analysis of alizarin red S indicated that the OD value in the M2-Exos group was dramatically higher than that in the M1-Exos group (Figure 5D). Moreover, as per RT-qPCR results, when exposed to M2-Exos, DPSCs obtained a markedly upregulated expression of odonto/osteogenic markers, including *ALP*, *DSPP*, *COL-1α1*, and *DMP-1*, *OCN*, *BMP-2*, and *RUNX-2* (Figure 5E). Generally, an opposite expression level was identified for M1-Exos, indicating their adverse role in odontoblastic differentiation.

### 3.6. Effect of M1-Exos and M2-Exos on Neurogenesis of DPSCs In Vitro

**Figure 6:**
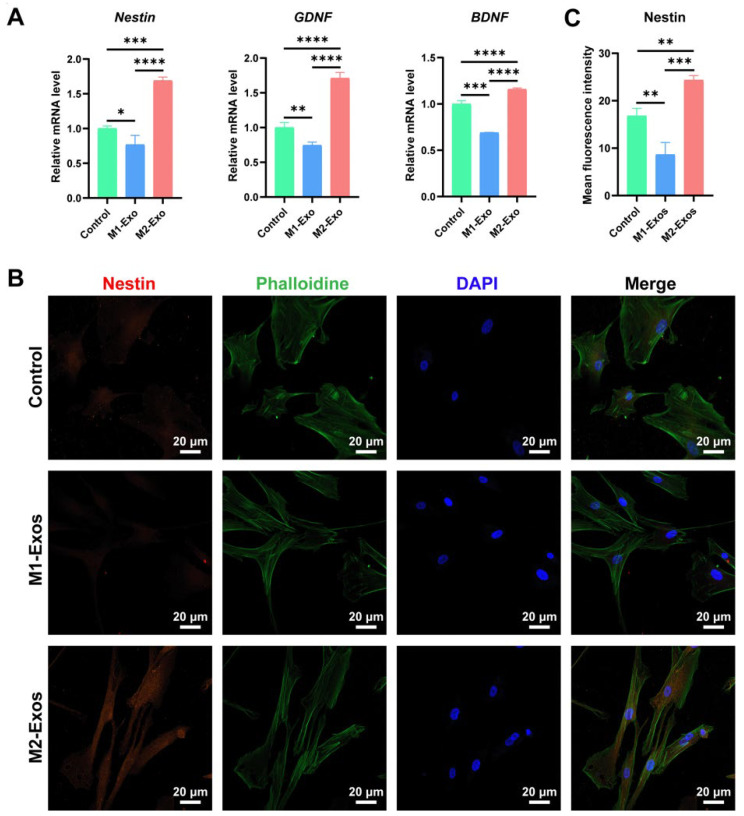
Effect of M1-Exos and M2-Exos on neurogenesis of DPSCs in vitro. (**A**) The expression of neurogenic markers varied according to M1-Exos and M2-Exos. In sharp contrast with M1-Exos, M2-Exos exhibited a robust potential to promote the expression of neurogenic genes, including *Nestin*, *GDNF*, and *BDNF*. (**B**) As revealed by immunofluorescence staining of Nestin, M1-Exos substantially inhibited Nestin expression (red fluorescence) in DPSCs (middle panel). However, M2-Exos permitted the co-expression of Nestin (red fluorescence), actin filament (green fluorescence), and nucleus (blue fluorescence) in DPSCs (lower panel). Scale bar, 20 μm. (**C**) Statistical analysis of mean fluorescence intensity of Nestin in DPSCs incubated with M1-Exos or M2-Exos. * *p* < 0.05, ** *p* < 0.01, *** *p* < 0.001, **** *p* < 0.0001.

In an attempt to investigate the role of M1-Exos and M2-Exos in the neurogenic differentiation of DPSCs, RT-qPCR and immunofluorescence staining were performed. As delineated by RT-qPCR results, M2-Exos produced an additive effect on the expression of neurogenesis-related markers, including *Nestin*, *GDNF*, and *BDNF* at the mRNA level in DPSCs (Figure 6A). On the contrary, M1-Exos were involved in the suppressed expression of these neurogenic genes (Figure 6A), highlighting the deleterious role of M1-Exos in neural repair and regeneration. Consistently, it was depicted through Nestin immunofluorescence staining that, under the influence of M2-Exos, Nestin expression was dramatically increased in DPSCs (Figure 6B, lower panel), which was in agreement with the statistical analysis of mean fluorescence intensity (Figure 6C). It was noteworthy that, in the presence of M1-Exos, DPSCs positive for Nestin were rarely identified (Figure 6B, middle panel), which was also verified by quantitative assessment (Figure 6C). Together, M2-Exos were intimately associated with the enhancement of the neurogenic differentiation of DPSCs in vitro.

### 3.7. Effect of M1-Exos and M2-Exos on Angiogenesis of DPSCs and HUVECs In Vitro and In Vivo

**Figure 7:**
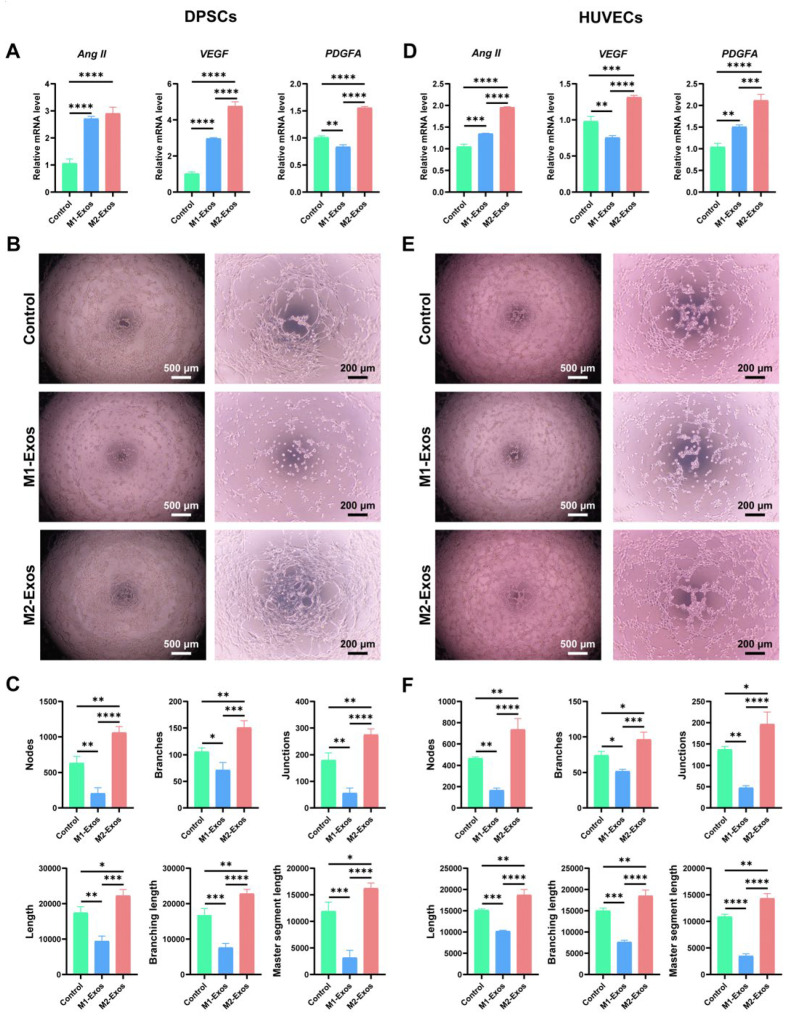
Effect of M1-Exos and M2-Exos on angiogenesis of DPSCs and HUVECs in vitro. (**A**,**D**) M2-Exos elicited a considerably enhancing impact on the expression of angiogenic markers at the mRNA level in DPSCs (**A**) and HUVECs (**D**). (**B**,**E**) By performing a tube formation assay in vitro, it was revealed that M1-Exos tremendously attenuated the angiogenic potential of DPSCs (**B**, middle panel) and HUVECs (**E**, middle panel), as evidenced by irregular and disordered structures. However, M2-Exos were instrumental in the tubular structure formation of DPSCs (**B**, lower panel) and HUVECs (**E**, lower panel), thus resulting in more abundant lumens and more continuous architecture. Scale bars, 500 μm and 200 μm (high magnification). (**C**,**F**) As confirmed by quantitative analysis, the application of M2-Exos remarkably enhanced the nodes, branches, length, and other angiogenic indexes of newly formed vascular-like structures in DPSCs (**C**) and HUVECs (**F**), thus exhibiting a beneficial effect on angiogenesis. On the contrary, M1-Exos negatively regulated nodes, branches, etc. * *p* < 0.05, ** *p* < 0.01, *** *p* < 0.001, **** *p* < 0.0001.

With the purpose of clarifying the effect of M1-Exos and M2-Exos on the angiogenesis of DPSCs and HUVECs in vitro, we conducted RT-qPCR and a tube formation assay. The results of the RT-qPCR demonstrated that the addition of M2-Exos considerably facilitated the expression of angiogenesis-associated mRNAs, including *ANG II*, *VEGF*, and *PDGFA* in DPSCs (Figure 7A) and HUVECs (Figure 7D). Notably, M1-Exos have shown a stimulatory effect on the expression of some genes, such as *ANG II*. However, a marked difference was determined between M1-Exos and M2-Exos. As indicated by the tube formation assay, M1-Exos severely restricted the formation of tubular structures in DPSCs (Figure 7B, middle panel) and HUVECs (Figure 7E, middle panel), which exhibited a messy and irregular appearance. Conversely, M2-Exos contributed to regularly arranged capillary-like tubules with more connections and lumens in DPSCs (Figure 7B, lower panel) and HUVECs (Figure 7E, lower panel). In accordance with these findings, the quantitative vascular index confirmed that M2-Exos robustly augmented the number of nodes, branches, length, etc (Figure 7C,F). In contrast, M1-Exos imposed a disadvantageous effect on these indexes, suggesting that M1-Exos negatively regulated tube formation in vitro. To sum up, M2-Exos not only had immense potential to promote the differentiation of DPSCs into vascular endothelial cells in vitro but could also functionalize as pericytes to induce the formation of vascular-like lumen structures in differentiated endothelial cells and existing HUVECs.

**Figure 8:**
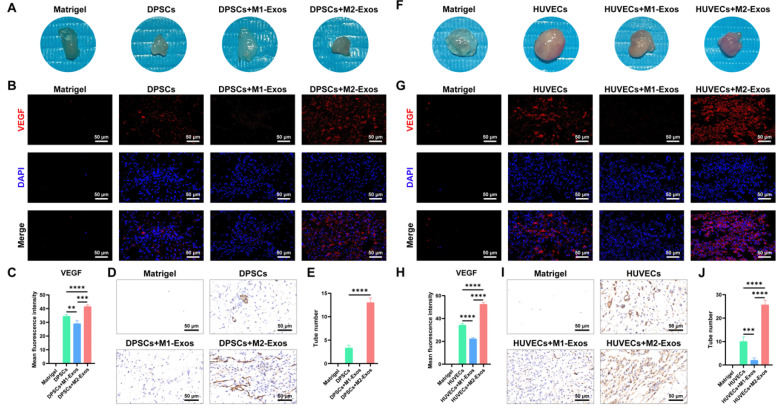
Effect of M1-Exos and M2-Exos on angiogenesis of DPSCs and HUVECs in vivo. (**A**,**F**) A general view of the Matrigel plugs dissected from the subcutaneous space of mice. The employment of M2-Exos conferred a reddish appearance on Matrigel plugs containing DPSCs (**A**) and HUVECs (**F**). However, Matrigel plugs were translucent or exhibited a reduced color under the influence of M1-Exos. (**B**,**G**) Representative immunofluorescence staining of VEGF. M1-Exos adversely affected the expression of VEGF in DPSCs (**B**) and HUVECs (**G**). Conversely, the application of M2-Exos contributed to the co-expression of VEGF (red fluorescence) and DAPI (blue fluorescence) in DPSCs (**B**, right-most panel) and HUVECs (**G**, right-most panel). Scale bar, 50 μm. (**C**,**H**) Statistical analysis of mean fluorescence intensity of VEGF in response to M1-Exos or M2-Exos in DPSCs (**C**) and HUVECs (**H**). (**D**,**I**) Representative immunohistochemical staining of CD31. M1-Exos considerably decreased the number of CD31^+^ vascular lumens in DPSCs (**D**) and HUVECs (**I**). In contrast, M2-Exos displayed an enhancing effect on the labeling of CD31^+^ vascular structures. Scale bar, 50 μm. (**E**,**J**) Statistical analysis of CD31^+^ tube structures formed in DPSCs (**E**) and HUVECs (**J**). ** *p* < 0.01, *** *p* < 0.001, **** *p* < 0.0001.

Focused on the role of M1-Exos and M2-Exos in angiogenesis in vivo, we transplanted Matrigel plugs into the subcutaneous space of nude mice for 2 weeks. As evidenced by the general view, the dissected Matrigel plugs incorporating M2-Exos displayed a reddish appearance, whereas the other Matrigel plugs showed a reduced or even absent color (Figure 8A,F), indicating the robust pro-angiogenic potential of M2-Exos. When subjected to immunofluorescence staining of VEGF, Matrigel plugs containing M2-Exos contributed to a significantly increased expression of VEGF, which was co-localized with DAPI in DPSCs (Figure 8B, right-most panel) and HUVECs (Figure 8G, right-most panel). In contrast, M1-Exos dramatically diminished VEGF immunostaining in DPSCs and HUVECs, supporting the adverse role of M1-Exos in angiogenesis. Consistent with these findings, statistical analysis of mean fluorescence intensity further confirmed the pro-angiogenic capability of M2-Exos and the anti-angiogenic potential of M1-Exos (Figure 8C,H). Furthermore, by using immunohistochemical staining, CD31, a specific vascular endothelium marker, was extensively identified in M2-Exos-pretreated DPSCs (Figure 8D) and HUVECs (Figure 8I), attributable to the crucial pro-angiogenesis of M2-Exos. On the contrary, M1-Exos substantially decreased the number of CD31-labeled vascular tubes (Figure 8D,I), which was further verified by quantitative evaluation (Figure 8E,J). Therefore, as demonstrated by in vitro and in vivo results, M2-Exos possessed excellent pro-angiogenic potential, which could markedly enhance the endothelial cell differentiation of DPSCs and capillary-like structure formation of newly differentiated endothelial cells and HUVECs.

## 4. Discussion

Macrophages are essential immune cells that initiate and regulate inflammatory response and innate–adaptive immunity. More importantly, macrophages contribute to maintaining and restoring tissue homeostasis within the body. When exposed to trauma or bacterial infection that results in the disturbance of the homeostatic microenvironment, macrophages can perform distinct biological functions, which will be either destructive or protective [24]. In accordance with their functional diversity, macrophages have exhibited phenotypic heterogeneity [25]. Depending on the versatile environmental stimuli, macrophages are classically polarized into pro-inflammatory M1 phenotypes or alternatively activated into anti-inflammatory M2 phenotypes. M1 macrophages possess a central role in mediating inflammatory cascade reactions and promoting adaptive immune responses by secreting pro-inflammatory cytokines, such as TNF-α. Conversely, protective M2 macrophages produce anti-inflammatory cytokines, such as IL-10, to switch off deleterious inflammatory processes and launch tissue remodeling and regeneration [26]. It has been confirmed that the polarization of M1 and M2 is significantly associated with pulpitis regression. The enhanced M1 polarization would exacerbate pulpitis progression [27], while M2 macrophages exerted a pivotal impact on the attenuation of reversible pulpitis and recovery of pulp tissue [28]. Concentrating on the relationship between macrophage phenotypes and nerve regeneration, Gao et al. [29] found that in the early acute stage of pulpitis, the ratio of M2 to M1 macrophages was considerably reduced in trigeminal ganglion. However, as pulp inflammation subsided, this ratio was reversed entirely, indicating that the phenotypic transformation of macrophages from M1 to M2 facilitated nerve repair in pulpitis. Aimed at enhancing tertiary dentinogenesis, Neves et al. [30] observed in a mouse pulp exposure model that macrophage depletion remarkably inhibited the formation of reparative dentin. In addition, the transition of reparative M2 macrophages from the M1 state accelerated local stem cell activation, thereby resulting in an elevated capacity for dentin repair. Collectively, these findings emphasize that macrophages, predominantly M2 macrophages, present immense potential to signal an advanced reconstruction of the pulp–dentin complex. As documented by Gu et al. [31] through a rat pulpotomy model, 3 days after implantation of hydrogel scaffolds loaded with rat bone marrow MSCs (BMMSCs) into the pulp chamber, no pulp-like tissue was observed, and the infiltration of macrophages was dominated by the M1 type. However, it is noteworthy that, 14 days later, regularly arranged pulp-like tissue that mimicked native pulp tissue and a distinctive dentin bridge could be identified in the pulp cavity. Intriguingly, the macrophage phenotype has been revealed to undergo an appropriate and favorable switch from M1 to M2 during pulp–dentin complex regeneration, leading to the predominance of M2 macrophages, which shared a similar distribution pattern with original pulp tissue. Hence, in clinical scenarios, stimulating M2 macrophage polarization by applying immunomodulatory biomaterials or biofactors has shown remarkable therapeutic value in alleviating pulpitis and achieving complete pulp–dentin complex regeneration. Nonetheless, the detailed mechanisms underlying M2 macrophage-mediated pulp tissue remodeling remain to be further illuminated. In view of this, an in-depth study was conducted by Park et al. [32] and indicated that M2 macrophage-derived conditioned medium (M2-CM) significantly enhanced ALP activity, *DSPP* expression, and the mineralized nodule accumulation of human dental pulp cells (hDPCs), highlighting that paracrine signaling from M2 macrophages can strengthen the odonto/osteogenic differentiation potential of hDPCs. Consistent with the above findings, Zhou et al. [33] recently have shown that M2-CM markedly promoted the proliferation, migration, and odonto/osteogenic differentiation of DPSCs in vitro. Moreover, after subcutaneous transplantation of DPSC sheets encapsulated into human tooth root fragments for 8 weeks, M2-CM induced the formation of functional dental pulp-like tissue, which was characterized by orderly arranged collagen fibers and a layer of odontoblast-like cells with their cytoplasmic processes extending into dentinal tubes. Notably, as one of the major active ingredients of paracrine effects, the roles and hidden mechanisms of exosomes derived from different subtypes of macrophages, especially M2 macrophages, in pulp–dentin complex regeneration are still elusive and thus warrant more profound exploration.

Exosomes can mediate intercellular communication by transferring a variety of biologically active substances, including RNA, proteins, and lipids, which are essential for maintaining normal cellular physiological activities and influencing pathological processes. As a result, exosomes have gradually become an ideal transport vehicle for delivering regulatory components to target cells [34]. In recent years, a growing amount of research suggests that exosomes represent a novel and desirable strategy for improving pulp–dentin complex regeneration. As previously discussed, 50 μg/mL exosomes released by stem cells from apical papilla (SCAP-Exos) considerably elevated the expression level of DSPP and the deposition of mineral nodules in BMMSCs [35]. Additionally, after being introduced into mouse subcutaneous space, SCAP-Exos contributed to the generation of blood vessel-enriched pulp tissue and overt polarized odontoblast-like cells that aligned to the newly formed continuous dentin layer, indicative of the promising employment of SCAP-Exos in regenerative endodontic practice. According to Wang et al. [36], exosomes derived from Schwann cells (SC-Exos) were capable of enhancing the proliferation, migration, and odonto/osteogenic differentiation of DPSCs and BMMSCs, as well as neurite outgrowth and vascular formation in vitro. As verified in vivo, SC-Exos successfully initiated the recruitment of endogenous stem cells and facilitated the regeneration of the pulp–dentin complex, which comprised dentin-like hard tissue, odontoblast-like cell layer, vascular structures, and neural tissue, shedding light on the immense potential of exosomes for engineering functional pulp tissue. Meanwhile, it is worth noting that the contents and functions of exosomes generally reflect the biological characteristics of their parental cells, thus mediating unique intercellular communication and accomplishing versatile therapeutic outcomes [37]. In line with this, Hu et al. [38] have elaborated that, in comparison with exosomes obtained from normal DPSCs (DPSC-Exos), exosomes derived from odontogenic DPSCs could downregulate the inhibitory molecule LTBP1 in DPSCs via transporting miR-27a-5p and activating the TGF-β1/Smads signaling pathway, which consequently promoted the odontogenic differentiation of DPSCs, as revealed by the extraordinarily augmented expression of the odontogenic markers dentin salivary protein (DSP), DMP-1, ALP, and RUNX-2. On the other hand, from the perspective of angiogenesis, Liu et al. [23] demonstrated that compared to exosomes secreted by normal stem cells from human exfoliated deciduous teeth (SHED), exosomes that originated from SHED cultured under hypoxic conditions (SHED-H-Exos) dramatically enhanced HUVEC proliferation and migration, as well as tubular structure formation in vitro and in vivo by conveying let-7f-5p and miRNA-210-3p, paving the way for developing SHED-H-Exos as a superior therapeutic approach in advancing pulp angiogenesis. In addition to odontogenic and hypoxic preconditioning, LPS stimulation has also been exploited as a desirable inducer of DPSCs to boost the odontogenic, angiogenic, and neurogenic capabilities of their exosomes, which orchestrate orthotopic regeneration of the whole functional pulp–dentin complex [39]. Collectively, more intensive attention should be paid to optimizing the therapeutic efficacy of exosomes in regenerative endodontics by culturing their parent cells under conducive circumstances. Considering the critical role of M2 macrophages in eradicating pulpitis and reconstructing the pulp–dentin complex, M2-Exos may open up a prospective avenue for administering regenerative endodontic procedures. To address this issue, we first prepared M1-Exos and M2-Exos using differential centrifugation and validated that M1-Exos and M2-Exos possessed typical round cup-shaped morphologies with average diameters of around 140 nm. Furthermore, the exosome-specific marker proteins TSG101 and CD63 were ubiquitously expressed on M1-Exos and M2-Exos, supporting the successful and effective isolation of exosomes in our study.

Selecting the appropriate stem cell type is a vital prerequisite for promoting tissue regeneration and repair. Since first discovered by Gronthos et al. [40] in 2000, DPSCs with robust self-renewal and multilineage differentiation potential have been extensively employed as seeding cells for oral tissue engineering [41]. Indeed, accumulating evidence has delineated that DPSCs represent an ideal stem cell source for pulp–dentin complex regeneration [38,42,43]. In our study, DPSCs extracted from healthy pulp tissue have exhibited the biological characteristics of MSCs, including plastic adherence, spindle-like morphology, and specific surface markers, as well as osteogenic, adipogenic, and chondrogenic differentiation capacity, consistent with previous studies. Since stem cell transplantation faces multiple obstacles in clinical translation, such as complex cell processing, improper immune rejection, distinctive social ethics, and strict political supervision, cell homing has been recognized as a more simple and feasible strategy for regenerative endodontics. This method depends on signaling molecules incorporated in scaffold materials to recruit endogenous stem cells, such as DPSCs and SCAP, and commence subsequent biological events [44,45]. With the aim of investigating the role of macrophage polarization in spinal cord injury, Li et al. [46] elucidated in vitro that the polarization of M2 macrophages substantially fostered the expression of PDGFB. Additionally, M2-CM elicited a profoundly facilitating effect on the migration of platelet-derived growth factor receptor β (PDGFRβ) positive pericytes, which could be impeded by a specific PDGFRβ inhibitor, indicating that M2 macrophage-secreted PDGFB may participate in pericyte migration by the PDGFB/PDGFRβ pathway. Notably, in addition to soluble factors, Zhu et al. [47] reported that M2-Exos robustly inhibited miR-493-3p secretion and activated the Akt/mTOR signaling pathway in human dermal fibroblast (HDFs) by transferring lncRNA-LINC01605, thereby enhancing the proliferation, migration, and invasion of HDFs. Taken together, these findings confirm the paramount impact of the M2 secretome on directing cell migration and accelerating tissue regeneration and raise the question of whether M2-Exos inspire the migratory and proliferative potential of DPSCs and HUVECs, thus contributing to cell homing-induced endogenous pulp–dentin complex regeneration. In light of this, we signified in the current study that M1-Exos and M2-Exos could be efficiently internalized by DPSCs and HUVECs, which expands their application in regulating the biological behaviors of DPSCs and HUVECs. More importantly, in direct contrast to M1-Exos, which imposed a dramatically inhibitory impact on cell functions, M2-Exos positively regulated the migration and proliferation of DPSCs and HUVECs, laying a solid foundation for further exploring the multifunctional value of M2-Exos and providing an innovative idea to pursue cell homing-based RET.

The ultimate goal of pulp–dentin complex regeneration is to achieve complete structural regeneration of tubular dentin and pulp tissue and to rebuild nutritional, sensory, and other functions. To this end, we have evaluated the effects of M1-Exos and M2-Exos on the odonto/osteogenic, neurogenic, and angiogenic differentiation of DPSCs. The generation of tubular dentin creates conditions for accommodating odontoblastic protrusions and dentinal fluid, which in turn promotes the conduction of external signals, such as temperature and mechanical stimuli. Encouragingly, the results of our study indicated that as opposed to M1-Exos, which considerably suppressed ALP staining and the mineralizing competency of DPSCs, the employment of M2-Exos displayed a distinct promoting efficacy on ALP performance and orange–red mineral nodule deposition. Consistently, the introduction of M2-Exos not only remarkably increased the expression of odonto/osteogenic genes, including *ALP*, *BMP-2*, *OCN*, and *RUNX-2*, but also definitely enhanced the level of the pivotal odontogenic markers *DSPP*, *DMP-1*, and *COL-1α1*. Representing a terminal differentiation marker of odontoblasts, *DSPP* plays crucial roles in dentin development [48]. Soon after its production, DSPP can be cleaved into two proteins, namely, DSP and dentin phosphoprotein, which initiate dentin mineralization and account for the maturation of dentin, respectively. DMP-1 is essential for phosphate metabolism and orchestrating mineral matrix deposition [49]. Meanwhile, type I collagen constitutes the most predominant component of the organic matrix in dentin [50]. Therefore, it can be tentatively concluded that M2-Exos are capable of accelerating odontoblast differentiation and the maturation of DPSCs in an early stage, which underlines the enormous potential of M2-Exos in inducing the formation of tubular dentin. Currently, there is still controversy concerning whether M1 macrophages stimulate stem cell differentiation and mineralization. Several studies have proven that M1-Exos presented an adverse effect on cell mineralization [51,52]. This is in agreement with our findings, which indicated that M1-Exos significantly inhibited ALP staining, alizarin red S staining, and the odonto/osteogenic gene expression of DPSCs. On the contrary, Liu et al. [53] revealed in a recent study that M1-Exos could facilitate the osteogenesis of BMMSCs by transferring miRNA-21a-5p during early inflammation. This inconsistency can be attributed to disparities in cell lineage and culture conditions, as well as diverse concentrations of exosome action. In particular, the exosome concentration employed by Liu et al. [53] was 1 μg/mL. As depicted by Yang et al. [54], low concentrations of exosomes could activate the autophagy pathway in BMMSCs under mild inflammatory environments, consequently ameliorating the mineralization process. In this regard, further studies are required to verify whether M1-Exos possess a beneficial effect on the odontogenic differentiation of DPSCs and to determine the superior concentration that accomplishes the goal of tubular dentin formation.

Complete pulp–dentin complex regeneration is inseparable from the restoration of neurosensory function. The phenotype of macrophages at the site of nerve injury drastically influences the outcomes of nerve regeneration. Reportedly, M1 macrophages intensified the inflammatory response and aggravated nerve injury [55]. Conversely, M2 macrophages could attenuate the inflammatory response, which enhanced nerve regeneration [56]. Simultaneously, Huang et al. [57] pointed out that triggering M1 to M2 conversion could accelerate peripheral nerve repair, which suggests an intimate correlation between the polarization of M2 macrophages and the achievement of nerve regeneration. As revealed by Zhan et al. [58], microvesicles secreted by M2 macrophages polarized from THP-1 cells dramatically promoted SC infiltration and axon elongation in vivo, thus providing a novel insightful direction for seeking nerve regeneration. Herein, it is worth mentioning that in our study, the M2-Exos stimulation of DPSCs gave rise to a substantially upregulated expression of neurogenic genes, including *BDNF*, *GDNF*, and *Nestin*. As a well-recognized neural precursor cell marker, *Nestin* exhibits an essential role in improving their renewal, survival, and proliferation potential [59]. For this reason, we implemented immunofluorescence staining of Nestin and confirmed that, consistent with the RT-qPCR results, M2-Exos considerably augmented the fluorescence expression of Nestin in DPSCs, which could be attributable to the robust neurogenic property of M2-Exos. In striking contrast, the neuroinhibitory effect of M1-Exos was very noticeable. Hence, the critical role of M2-Exos in boosting DPSC neurogenic differentiation in vitro is anticipated to improve nerve repair in regenerating dental pulp tissue, which deserves further exploration.

Angiogenesis will provide necessary oxygen and nutrients to the nascent pulp tissue and is fundamental for the proper regeneration of the pulp–dentin complex [60]. Recently, Song et al. [61] clarified that platelet-derived exosomes would drive phenotypic switching of M1 to M2 macrophages through activating the TGF-β pathway, thereby promoting angiogenesis and collagen synthesis, which confer marked advantages to M2 macrophages for the healing of traumatic injuries. A growing number of studies have shown that M2-Exos could enhance angiogenesis by delivering miRNA-942 [62], while M1-Exos deleteriously repressed angiogenesis [63]. According to our RT-qPCR results, M2-Exos remarkably facilitated the expression of the angiogenic markers *ANG II*, *VEGF*, and *PDGFA* in DPSCs. Notably, M1-Exos also elicited a stimulatory effect on the expression of *ANG II* and *VFGF* in DPSCs. Considering that exosomes can encapsulate parent cell-derived soluble cytokines during their biogenesis [64,65], it is speculated that M1-Exos may exert their pro-angiogenic competency by virtue of those incorporated angiogenic factors, such as ANG II. However, blood vessels developed under the instruction of a limited amount of pro-angiogenic cytokines are immature and susceptible to degradation [65]. This is also illustrated by the scattered and irregular vascular-like structures produced by DPSCs after the administration of M1-Exos in our tube formation assay. On the contrary, the distribution of tubular structures generated by DPSCs after M2-Exos pretreatment was outstandingly favorable, as evidenced by elevated numbers of nodes, branches, junctions, etc. Meanwhile, after the subcutaneous implantation of Matrigel plugs containing M2-Exos and DPSCs, more reddish capillary-like networks, significantly higher VEGF expression, and widely spread CD31^+^ tubular lumens further demonstrated the tremendous potential of M2-Exos in driving DPSC vascular endothelial cell differentiation and angiogenesis. In order to elucidate whether M2-Exos perform pericyte functions to regulate angiogenic sprouts of existing vascular endothelial cells, we specifically conducted in vitro and in vivo angiogenesis experiments using HUVECs. Intriguingly, resembling pericytes, M2-Exos profoundly promoted the angiogenic capability of HUVECs, as ascertained by the enhanced expression of angiogenic genes, continuous lumenogenesis in vitro, and abundant vascular network rebuilding in vivo. Accordingly, as validated by DPSC- and HUVEC-induced angiogenesis, the introduction of M2-Exos offers promising prospects to expand the laboratory and clinical reach of functional pulp–dentin complex regeneration.

As a shortcoming of the present study, the molecular mechanisms underlying the multifaceted roles of M2-Exos in ameliorating DPSC and HUVEC functions have not been discussed. To date, a variety of enriched proteins and RNAs, such as lncRNAs, mRNAs, miRNAs, and circRNAs, have been established to be responsible for exosomal biological effects [13]. As suggested by Li et al. [66] via proteomics analysis, exosomes from hypoxia preconditioned DPSCs displayed a remarkably promoting impact on the migration, proliferation, and vascular-like structure formation of HUVECs by transferring lysyl oxidase-like 2. Furthermore, it has been verified that M2-Exos were capable of triggering vascular regeneration by transporting the ubiquitin thioesterase otulin protein through the initiation of the Wnt/β-catenin pathway, which was instrumental to neurological functional recovery post spinal cord injury [67]. Aside from functional proteins, Li et al. [68] have revealed that DPSC-Exos facilitated MSC migration and osteoblastic differentiation by delivering lncRNA-Ankrd26, which activated the miR-150/TLR4 signaling pathway. In addition, the lncRNA-LOC103691165 of macrophage-derived exosomes has been identified to enhance the osteogenic capacity of BMMSCs [69]. Moreover, exosomal miRNAs also exert crucial effects on both osteogenic and angiogenic differentiation. M1-Exos containing miRNA-155 inhibited the BMP signaling pathway and consequently reduced osteogenesis, while miRNA-378a in M2-Exos stimulated BMP signaling and thus promoted osteogenic behaviors [52]. From the perspective of angiogenesis, Gollmann-Tepeköylü et al. [70] elaborated that exosomes collected from shock-wave-treated HUVECs facilitated cellular proliferation and angiogenesis by initiating Akt/Erk signaling through the transfer of miRNA-19a-3p, resulting in substantially improved ischaemic myocardial function. Collectively, these findings corroborate the diverse roles of functional proteins and RNAs packaged into exosomes in tissue regeneration. Inspired by this notion, we hypothesize that M2-Exos could serve as appropriate vehicles to deliver proteins and/or protein/RNA complexes, positively modulating cellular proliferation and migration, as well as odonto/osteogenic, neurogenic, and angiogenic differentiation. In subsequent studies, we will conduct RNA sequencing and proteomics analysis to disclose the detailed mechanisms by which M2-Exos contribute to the regeneration of the pulp–dentin complex.

## 5. Conclusions

To the best of our knowledge, this study demonstrated for the first time that M2-Exos were beneficial in inducing the migration and proliferation of DPSCs and HUVECs, as well as the multilineage differentiation of DPSCs and the angiogenesis of HUVECs, supporting the multifunctional capacity of M2-Exos. The implementation of the current study has complemented the mechanism of action of M2 macrophages in ameliorating reversible pulpitis and promoting restorative dentin regeneration by delivering exosomes under pathological conditions. More importantly, this study provides crucial therapeutic clues of an endogenous cell homing strategy for achieving complete functional regeneration of the pulp–dentin complex in vivo. Taken together, the multifunctional M2-Exos will open up a new way with broad prospects for promoting the clinical translation of regenerative endodontic treatment.

## Data Availability

The data that support the findings of current research are openly available in the article. Raw and derived data underlying the findings of current research are available from the corresponding author upon reasonable request.

## Supplementary Materials

The following supporting information can be downloaded at: https://www.mdpi.com/article/10.3390/biomedicines12020441/s1; Table S1: Reverse transcription reaction system; Table S2: Primer sequences required for RT-qPCR analysis; Table S3: RT-qPCR reaction system.
