# Supplementary material for: Multifunctional Exosomes Derived from M2 Macrophages with Enhanced Odontogenesis, Neurogenesis and Angiogenesis for Regenerative Endodontic Therapy: An In Vitro and In Vivo Investigation
Source: Biomedicines. 2024 Feb 16;12(2):441. doi: 10.3390/biomedicines12020441 (PMC10886856; doi:10.3390/biomedicines12020441)
Supplement: Supplementary file 1 [file biomedicines-12-00441-s001.zip › biomedicines-2850426-supplementary.pdf]

Multifunctional Exosomes Derived from M2 Macrophages with Enhanced Odontogenesis, Neurogenesis and Angiogenesis for Regenerative Endodontic Therapy: An In Vitro and In Vivo Investigation

TABLE S1 Reverse transcription reaction system

Reagent	Amount
Total RNA	500 ng
5× prime script RT mix	2 μL
RNase free water	to 10 μL
Total	10 μL

TABLE S2 Primer sequences required for RT-qPCR analysis

Gene	primer	Sequence (5′- 3′)
β-actin	Forward	TGGCACCCAGCACAATGAA
	Reverse	CTAAGTCATAGTCCGCCTAGAAGCA
iNOS	Forward	GCTCTACACCTCCAATGTGACC
	Reverse	CTGCCGAGATTTGAGCCTCATG
IL-6	Forward	AGACAGCCACTCACCTCTTCAG
	Reverse	TTCTGCCAGTGCCTCTTTGCTG
CD86	Forward	CCATCAGCTTGTCTGTTTCATTCC
	Reverse	GCTGTAATCCAAGGAATGTGGTC
TNF-α	Forward	CTCTTCTGCCTGCTGCACTTTG
	Reverse	ATGGGGCTACAGGCTTGTGTCACTC
IGF	Forward	CTCTTCAGTTCGTGTGTGGAGAC
	Reverse	CAGCCTCCTTAGATCACAGCTC
TGF-β	Forward	TACCTGAACCCGTGTTGCTCTCTC
	Reverse	GTTGCTGAGGTATCGCCAGGAA
VEGF	Forward	TTGCCTTGCTGCTCTCTACCTCCA
	Reverse	GATGGCAGTAGCTGCGCTGATA
CD163	Forward	CCAGAAGGAACTTGTAGCCACAG
	Reverse	CAGGCACCAAGCGTTTTGAGCT
ALP	Forward	ATGGGGATGGGTGTCTCCACA

	Reverse	CCACGAAGGGGAACTTGTC
RUNX-2	Forward	TGGTTACTGTCATGGCGGGTA
	Reverse	TCTCAGATCGTTGAACCTTGCTA
BMP-2	Forward	CGTCAAGCCAAACACAAACAG
	Reverse	GCCACAATCCAGTCATTCCAC
OCN	Forward	GGCGCTACCTGTATCAATGGG
	Reverse	GTGGTCAGCCAACTCGTCA
COL-1α1	Forward	GATTCCCTGGACCTAAAGGTGC
	Reverse	AGCCTCTCCATCTTTGCCAGCA
DMP-1	Forward	CCAATGGGCTACTCTGCTCACATG
	Reverse	AAGCCATCTCGGACCTCCACAT
DSPP	Forward	TGGCGATGCAGGTCACAAT
	Reverse	CCATTCCCACTAGGACTCCCA
Nestin	Forward	GAAGGGCAATCACAACAGGTG
	Reverse	GGGGCCACATCATCTTCCA
GDNF	Forward	GCAGACCCATCGCCTTTGAT
	Reverse	CCACACCTTTTAGCGGAATGC
BDNF	Forward	TAACGGCGGCAGACAAAAAGA
	Reverse	TGCACTTGGTCTCGTAGAAGTAT
ANG II	Forward	CCTCCATGCCAGTACCGAG
	Reverse	GGACGACGGAAAATTGACTGA
PDGFA	Forward	GCAAGACCAGGACGGGTCATTT
	Reverse	GGCACTTGACACTGCTCGT

TABLE S3 RT-qPCR reaction system

Reagent	Amount
SYBR Green	10 μL
Template	100 ng
Primer F (10 μM)	0.4 μL
Primer R (10 μM)	0.4 μL
RNase free water	to 20 μL
Total	20 μL
